# Spectro-Temporal Weighting of Loudness

**DOI:** 10.1371/journal.pone.0050184

**Published:** 2012-11-28

**Authors:** Daniel Oberfeld, Wiebke Heeren, Jan Rennies, Jesko Verhey

**Affiliations:** 1 Department of Psychology, Section Experimental Psychology, Johannes Gutenberg-Universität Mainz, Mainz, Germany; 2 Department of Experimental Audiology, Otto-von-Guericke University Magdeburg, Magdeburg, Germany; 3 Hearing, Speech and Audio Technology, Fraunhofer-Institut für Digitale Medientechnologie IDMT, Oldenburg, Germany; UNLV, United States of America

## Abstract

Real-world sounds like speech or traffic noise typically exhibit spectro-temporal variability because the energy in different spectral regions evolves differently as a sound unfolds in time. However, it is currently not well understood how the energy in different spectral and temporal portions contributes to loudness. This study investigated how listeners weight different temporal and spectral components of a sound when judging its overall loudness. Spectral weights were measured for the combination of three loudness-matched narrowband noises with different center frequencies. To measure temporal weights, 1,020-ms stimuli were presented, which randomly changed in level every 100 ms. Temporal weights were measured for each narrowband noise separately, and for a broadband noise containing the combination of the three noise bands. Finally, spectro-temporal weights were measured with stimuli where the level of the three narrowband noises randomly and independently changed every 100 ms. The data consistently showed that (i) the first 300 ms of the sounds had a greater influence on overall loudness perception than later temporal portions (primacy effect), and (ii) the lowest noise band contributed significantly more to overall loudness than the higher bands. The temporal weights did not differ between the three frequency bands. Notably, the spectral weights and temporal weights estimated from the conditions with only spectral or only temporal variability were very similar to the corresponding weights estimated in the spectro-temporal condition. The results indicate that the temporal and the spectral weighting of the loudness of a time-varying sound are independent processes. The spectral weights remain constant across time, and the temporal weights do not change across frequency. The results are discussed in the context of current loudness models.

## Introduction

Loudness is the sensation which is most closely related to the intensity of a sound. However, loudness also depends on other characteristics of the sound such as its duration or its spectral content. Several studies demonstrated that at equal sound pressure level a broadband signal is usually louder than a narrow-band signal (e.g., [1], [2], [3], [4], [5], [6], [7], [8], [9]). This effect is commonly referred to as *spectral loudness summation*. It can be accounted for by assuming that the auditory system analyzes the incoming sound with a bank of overlapping band-pass filters (critical bands) followed by a compressive nonlinearity and a summation across critical bands [10], [11], [12].

Apart from the integration across frequency, the auditory system also seems to integrate over time: The level of a short signal is usually higher than the level of an equally loud long signal with the same spectrum [13], [14], [15], [16], [17]. This temporal integration is usually accounted for by assuming a leaky integrator as a temporal integration stage and a decision device that uses the maximum or a percentile of the output of this stage. Current elaborate loudness models include both a spectral and a temporal stage of the kind described above to account for loudness of time-varying sounds like speech or the noise of a vehicle passing by (e.g., [18], [19]).

The traditional technical measures for the loudness of time-varying sounds, for example *L_Aeq_* (the A-weighted energy-equivalent sound pressure level) or *N*_5_ (the 95^th^ percentile of the loudness distribution) (cf. [20], [21]) which are used in standards on noise assessment (e.g., [22]), are based on the assumption that all temporal portions of the sound contribute in the same way to overall loudness. More precisely, two temporal portions with identical spectrum and level have the same impact on *L_Aeq_* and similar measures, regardless of their temporal position within the sound (e.g., beginning versus end). However, this assumption was recently challenged by studies of temporal weights in loudness judgments. These studies investigated the importance of different temporal segments for global loudness judgments based on stimuli with only small, random level fluctuations (e.g., [23], [24], [25], [26]). Using methods from so-called *molecular psychophysics* [27], [28], [29], perceptual temporal weights were obtained at high temporal resolution. The term *molecular psychophysics* [27] refers to trial-by-trial analyses that provide information about the relation between a stimulus feature (e.g., the level of one component of a multitone stimulus) and the response of the listener (e.g., “signal present” versus “signal absent”). These methods, for which the alternative terms *perceptual weight analysis* or *behavioral reverse correlation* have been used, typically impose random trial-by-trial variation on the stimulus components. For example, in a study measuring temporal weights (e.g., [30], [31]), the stimulus might consist of 10 temporal segments. All segments are presented with a mean level of 60 dB SPL, but the levels are randomly and independently varied from trial to trial. Correlational or regression analyses are then used to estimate the impact of the variation of each individual stimulus feature on a behavioral or neural response (for a detailed explanation see [25], [30]). These weights represent the influence of the level of individual temporal portions of a sound on the loudness of the sound as a whole (*global loudness*). More specifically, the weights show how strongly the global loudness changes when the level of a temporal portion of the sound is changed. Studies on the temporal weighting of loudness consistently showed that the first 100–300 ms receive a higher weight than later portions of the stimulus [24], [25], [30], [31]. This means that, for example, a 1 dB increase in the level of the first 100 ms of the sound causes a stronger increase in global loudness than a 1 dB increase in the level of the final 100 ms. This *primacy effect* (highest weight assigned to the beginning of a sound) only occurs for signals with a similar level across the complete duration of the signal (i.e., when the level does not change over time). A *delayed primacy effect* is observed when the level of the first part of the signal is reduced compared to the rest of the stimulus [25], [30]. In this case, the first temporal segment presented at the full level receives the highest weight. Results of Oberfeld and Plank [30] suggest that this effect can be explained by attention to the loudest elements, which was proposed as an explanation for higher weights observed for loud elements, even if these elements provided less reliable information than softer elements [32], [33], [34]. Some studies also showed a recency effect, i.e., higher weights on the last 100–200 ms of the signal [23], [24]. However, this effect appears to be considerably smaller than the primacy effect and was non-significant in most studies (e.g., [26], [31]). In any case, the data show that not all temporal portions contribute to loudness to the same extent, contrary to what is assumed by technical measures of loudness such as *L_Aeq_* or *N*_5_ [35], [36], [37], which are used in standards on noise assessment like ISO 1996 [22]. It is interesting to note that, according to simulation results by Pedersen [38] and unpublished simulations by one author (DO), recent models for the loudness of time-varying sounds [18], [19] (one of them is used in a German standard, [21]) cannot account for the observed primacy effect, even though they include a temporal integration stage and some effects of temporal masking.

Apart from temporal weights in the processing of the level of the sound, spectral weights have been investigated for sounds without temporal variability. As for the temporal weights, these studies generally reported a non-uniform spectral weighting of auditory intensity [39], [40], [41], [42], [43], [44], for example higher weights on the lowest and/or highest frequency component than on the middle components.

Previous studies either considered the *temporal* weighting of loudness but did not look at *spectral* weights, or measured spectral weights but did not consider temporal aspects of loudness. Therefore, in previous experiments, the stimuli were constructed so that there was either only a variation in intensity across time (both within a trial and between trials), or only a variation across frequency (between trials).

Outside the laboratory, however, as real-world sounds like speech or traffic noise unfold in time, the energy in different spectral regions typically evolves differently. In the present study, we therefore estimated *spectro-temporal weights* for global loudness judgments by introducing independent temporal variations in level in different spectral regions within each stimulus. These spectro-temporal weights were compared to temporal weights and spectral weights measured for the same listeners.

## Materials and Methods

### Ethics Statement

The experiments were conducted according to the principles expressed in the Declaration of Helsinki. All listeners participated voluntarily after providing informed written consent. They received partial course credit or were paid for their participation. The study was approved by the ethical committee of the University of Oldenburg.

### Participants

Due to the considerable experimentation time required for each participant (15 sessions, see below), data were collected at two laboratories (Mainz and Oldenburg). In both laboratories, the same software code (MATLAB) was used for stimulus generation and for the control of the experiment. The apparatus was also virtually identical (for details see section Apparatus). In each laboratory, five listeners participated (Mainz: 1 male, 4 female, age 19–29 years. Oldenburg: 2 male, 3 female, 24–31 years). All reported normal hearing and no history of hearing disorders.

### Apparatus

The stimuli were generated digitally. In Mainz, they were played back via two channels of a RME ADI/S D/A converter (*f*_s_ = 44.1 kHz, 16-bit resolution), attenuated by TDT PA5 attenuators, and buffered by a TDT HB7 headphone buffer. In Oldenburg, they were played back via two channels of a RME ADI-8 PRO D/A converter and amplified by a TDT HB7. In both labs, the sounds were presented diotically via Sennheiser HDA 200 circumaural headphones calibrated according to IEC 318 [45] and free-field equalized according to IEC 389-5 and 389-8 [46]. The experiment was conducted in double-walled sound-insulated chambers. Listeners were tested individually.

### Stimuli and Conditions

**Figure 1:**
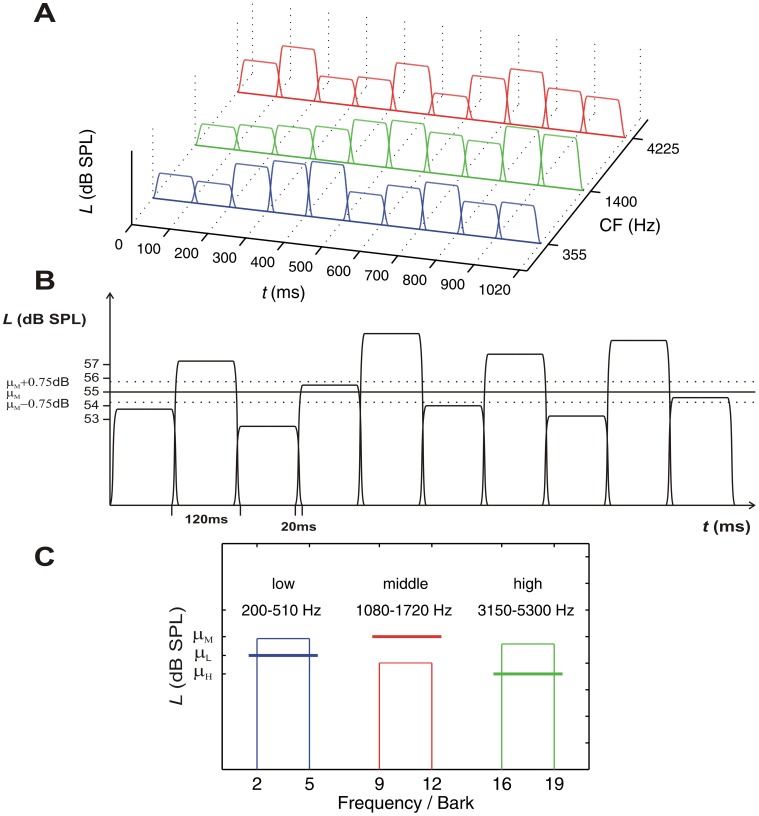
Stimulus with spectro-temporal variation. Panel A: In the spectro-temporal condition the stimulus consisted of three narrowband noises. For each noise band, ten temporal segments were presented. Independent and random level perturbations were imposed on the 3 (noise band) × 10 (segment) component levels. Panel B: temporal configuration for the mid-CF noise band. The 10 segment levels were drawn independently from a normal distribution with mean *µ_M_* = 55 dB SPL and a standard deviation of 2.5 dB. With identical probability, either 0.75 dB was subtracted from or added to each segment level, in order to create “soft” and “loud” trials (see text). For the low and high noise band the same temporal configuration was used. Panel C: Spectral configuration.

In the *spectro-temporal condition*, the stimuli consisted of 10 temporally overlapping noise segments with a total duration of 120 ms including 20 ms cos^2^-ramps at onset and offset (see **Figure 1**, Panel A). **Figure 1**, Panel B shows the temporal structure for a single frequency band. The effective duration of each segment was 100 ms. The segments had an overlap of 20 ms resulting in an overall stimulus duration of 1020 ms (effective duration was 1000 ms). Each temporal segment contained energy in three different frequency bands (see **Figure 1**, Panel C). The three frequency bands were 3.0 Bark wide to prevent strong level fluctuations that would be audible with smaller bandwidths. In the same vein, we presented Gaussian low-noise noises generated by means of two iterations [47], although this may not have further reduced audible fluctuations because the bandwidth of the stimuli exceeded the auditory filter bandwidth. The noise bands were separated by 4.0 Bark. The noise band with the low center frequency (CF) had cut-off frequencies of 200 Hz and 510 Hz (2.0 to 5.0 Bark) resulting in an arithmetic CF of 355 Hz. The cut-off frequencies for the other two noise bands were 1080 and 1720 Hz (9.0 to 12.0 Bark) for the middle and 3150 and 5300 Hz (16.0 to 19.0 Bark) for the high frequency band, corresponding to CFs of 1400 and 4225 Hz, respectively. The total bandwidth of signals containing all of the three noise bands was 17.0 Bark (23.67 CAM; [48]).

The middle frequency band was presented at a mean sound pressure level of *µ_M_* = 55 dB SPL. The mean level of the high (*µ_H_*) and the low frequency band (*µ_L_*) was selected individually on the basis of loudness matches (see *Methods*, subsection *Loudness matches*), so that all noise bands were equally loud. This is an important aspect of the design because there is evidence that louder elements receive higher weights [32], [33], [34], [49].

On each trial, the sound pressure level of each noise band was randomly selected for each temporal segment by drawing independently from a normal distribution with mean *µ_L_*, *µ_M_*, or *µ_H_* and standard deviation 2.5 dB. Thus, in the spectro-temporal condition the sound contained 30 components (noise band × segment) with levels independently and randomly selected. On each trial, 0.75 dB was added to or subtracted from the level of all components (with equal probability) in order to make it either a “loud” or a “soft” trial, respectively [25]. With a 1.5 dB difference between the level distributions of “loud” and “soft” trials we expected the sensitivity in the intensity identification task in terms of the area under the ROC curve (AUC; e.g., [50]) to be in the vicinity of 0.7, based on previous experience with this kind of task [30].

**Figure 2:**
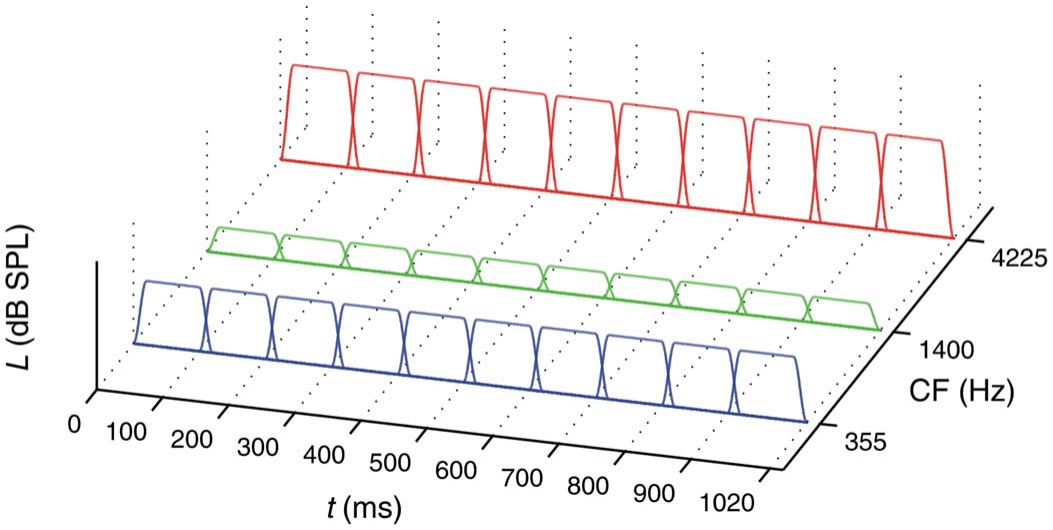
Stimulus presented in the spectral-weights condition.

The stimuli in the *spectral-weights condition*, the *broadband-noise condition*, and the *single-noise-band conditions* were constructed in the same way as for the spectro-temporal condition, but contained less components. The selection of the mean levels for the three noise bands, the random level perturbations, and the addition or subtraction of 0.75 dB were done exactly as for the spectro-temporal condition. In the *spectral-weights condition* (see **Figure 2**), there was spectral variation from trial to trial, but no temporal variation within a sound. In this condition, ten overlapping segments each with a duration of 120 ms were presented for each noise band, just as in the spectro-temporal condition. However, within each noise band the levels of the ten segments were identical.

**Figure 3:**
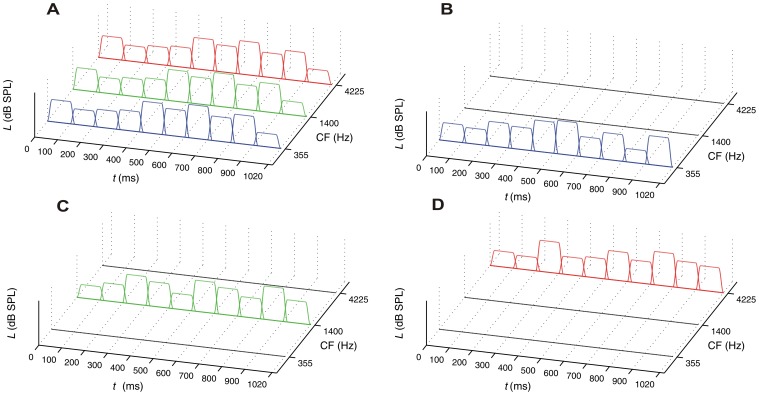
Stimuli with purely temporal variation. Panel A: broadband noise. Panel B: low-CF noise band. Panel C: mid-CF noise band. Panel D: high-CF noise band.

In the *broadband-noise condition* (see **Figure 3**, Panel A), each segment also comprised the three noise bands, but the levels of the three noise bands were perfectly correlated (*r* = 1.0) in each temporal segment. Thus, effectively a broadband noise varying in level across time was presented, and there was only temporal but no spectral variation (i.e., the spectral composition was identical for all temporal segments, and only the level of the segments varied). Finally, in the three *single-noise-band conditions* (see **Figure 3**, Panels B to D), each sound consisted of 10 temporal segments containing only the low-CF, middle-CF, or high-CF noise band. Again, there was only temporal but no spectral variation.

### Procedures

#### Loudness matches

Prior to the actual experiment, the three different band-pass noises were equalized in loudness for each listener. For that purpose, the loudness of each noise was matched to the loudness of the other two separately. Within each of the three pairs (low-mid, low-high, and mid-high), both of the noises once served as the reference stimulus (fixed level) and once as the test stimulus (level varied by the adaptive procedure, see below). Additionally, the test stimulus had an initial level of either +10 or −10 dB relative to the level of the reference stimulus, resulting in a total of twelve different adaptive tracks (noise pair × reference stimulus × initial level). To further reduce bias effects, the resulting twelve tracks were randomly interleaved (cf. [51], [52]). Three blocks were presented, each containing the twelve interleaved tracks. For each track, the loudness of the test stimulus was matched to that of the reference stimulus using an adaptive two-interval, two-alternative forced-choice procedure with a one-up, one-down rule [53], tracking the 50%-point of the psychometric function. On each trial, the listeners heard two sounds and indicated which one was louder by pressing the corresponding button on a computer keyboard. The test stimulus was presented either in interval 1 or in interval 2 with equal a priori probability. The presentation intervals were 1000 ms long and separated by 500 ms of silence. The reference stimulus had a fixed level of 55 dB SPL for the middle-CF noise. For the low-CF and the high-CF reference stimuli, the fixed levels were selected to give the same loudness as the middle-CF reference stimulus according to the loudness model of Chalupper and Fastl [19]. The corresponding levels were calculated as 57.0 dB SPL for the low-CF and 52.0 dB SPL for the high-CF reference stimulus. The level of the test stimulus was decreased if the listener indicated it to be louder than the reference. Otherwise it was increased. The initial increment or decrement in level was 8 dB, and this was halved after each upper reversal until a step size of 2 dB was reached. With this step size, the procedure was continued until another six reversals occurred. The mean of the levels at these final six reversals was used to calculate the level difference between the reference and the equally loud test stimulus. To adjust the noise bands to equal loudness in the main experiment, for each reference noise band the mean level difference between the reference and each test noise band across the two starting levels and the three measurement blocks was computed. Finally, the average of the matches across the three reference noise bands relative to the mid-CF noise band provided the level differences for equal loudness of the three noise bands.

#### Loudness identification task used for the estimation of weights

A one-interval absolute intensity identification task was used for estimation of the spectro-temporal weights [54]. On a four-point ordered rating scale, listeners indicated whether a loud or a soft sound had been presented, and at the same time expressed their confidence when making the decision. The scale comprised the response categories “Soft – rather sure”, “Soft – rather unsure”, “Loud – rather unsure”, and “Loud – rather sure” (in German: “Leise - eher sicher”, “Leise - eher unsicher”, “Laut - eher unsicher”, and “Laut - eher sicher”). Listeners responded by pressing the corresponding buttons on a keyboard. This rating scale including information about confidence was used in order to be able to construct ROC curves for estimating sensitivity. In this way, we avoided the necessity to make potentially unjustified assumptions about the form of the ROC curve, which would be necessary for using *d* ´ based on binary responses as a measure of sensitivity [50], [55]. Because the average overall loudness varied from block to block and was for example higher for the spectro-temporal condition than for the single noise-band conditions, listeners were instructed not to consider the loudness of sounds presented in previous blocks when classifying the sounds in the current block as either soft or loud. Note that the task of deciding whether a soft or a loud trial was presented can be described as a sample discrimination task [56], [57], [58]. No trial-by-trial feedback was provided.

Each experimental block contained only one of the six conditions (spectro-temporal condition, spectral weights condition, broadband noise condition, and the three single noise band conditions). 91 trials were presented per block. In the main part of the experiment (sessions 5 to 15, see below), each session comprised three blocks of the spectro-temporal condition, and one block of each of the remaining conditions (spectral weights condition, broadband noise condition, and the three single noise band conditions). The order of blocks was randomized, except that the spectro-temporal condition was not presented in consecutive blocks. Across the 11 sessions presenting the intensity identification task, 3003 trials were collected for each listener in the spectro-temporal condition, and 1001 trials in each of the remaining conditions. A higher number of trials was collected for the spectro-temporal condition because of the necessity to estimate a higher number of weights (30) than in the remaining conditions, where only 3–10 weights had to be estimated.

### Sessions

In session 1, practice blocks for all experimental tasks and conditions were run. In session 2, loudness matches were obtained for the three noise bands. Additionally, listeners received practice blocks in the identification task. In following sessions only the identification task was presented. Each session lasted about 50 minutes. Listeners participated in one or two sessions per day, separated by a pause of at least 30 minutes.

#### Weight estimation

The decision weights representing the importance of the 30 spectro-temporal components for the decision in the intensity identification task were estimated from the trial-by-trial data via multiple logistic regression [24], [25], [59], [60]. For the spectro-temporal condition containing three noise bands with 10 temporal segments each, the decision variable underlying the analysis is given by
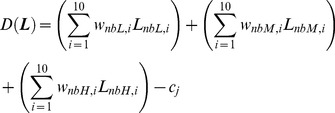
(1)where ***L*** is a vector of component levels, *L_nbL,i_* denotes the level of the noise band with the low CF in segment *i* (*i* = 1, …, 10), *w_nbL,i_* is the decision weight assigned to the level of this component, the indices *nbM* and *nbH* denote the noise band with the intermediate and the high CF, respectively, and *c_j_* is a constant representing the decision criterion for the *j* ^th^ of the four ordered response categories [24], [29], [61]. In other words, *D*(***L***) is a weighted average of the 30 (noise band × segment) independent component levels.

The decision model (Eq. (1)) assumes that, on a given trial, a listener responds that a loud rather than a soft sound was presented if *D*(***L***) >0. More precisely, as we have a four-category response variable *Y* we assumed a proportional-odds model [62] according to which
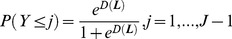
(2)where *J* is the number of ordered response categories. This model applies simultaneously to all *J* − 1 cumulative probabilities, and it assumes an identical effect of the predictors for each cumulative probability [61].

For the remaining conditions, the decision variable was constructed analogously to Eq. (1), but contained fewer components. For example, for the spectral weights condition there were only three component levels and three corresponding weights.

In the data analysis, the ordered categorical responses (“Soft – rather sure”, “Soft – rather unsure”, “Loud – rather unsure”, and “Loud – rather sure”) served as the dependent variable. The predictors (i.e., component levels) were entered simultaneously. The regression coefficients were taken as the decision weight estimates. For a given component (e.g., the level of the first segment of the low-CF noise), a regression coefficient equal to zero means that the component had no influence at all on the decision to judge the sound as being soft or loud. A regression coefficient greater than zero means that the probability of responding that the *loud* sound was presented increased with the sound pressure level of the given component. A regression coefficient smaller than zero indicates the opposite relation.

Due to the difference in mean level between “loud” and “soft” trials, the component levels were correlated. Therefore, separate logistic regression analyses were conducted for the “loud” trials where 0.75 dB had been added to all component levels, and for the “soft” trials where 0.75 dB had been subtracted (cf. [29]). This avoids potential problems with multicollinearity, although the multiple logistic regression procedure corrects for correlations between the covariates.

A separate logistic regression model was fitted for each combination of listener, condition, and trial type (“soft” versus “loud” trials). As we were interested in the *relative* contributions of the different components to the decision rather than in the absolute magnitude of the regression coefficients, the decision weights *w*_i_ were normalized for each fitted model such that the mean of their absolute values was 1.0 (see [40]), resulting in a set of relative decision weights for each listener, condition, and trial type (“soft” or “loud”).

A summary measure of the predictive power of a logistic regression model is the area under the receiver operating characteristic (ROC) curve [50], [63]. This measure provides information about the degree to which the predicted probabilities are concordant with the observed outcome (see [26] for details). Areas of 0.5 and 1.0 correspond to chance performance and perfect performance of the model, respectively. Across the 120 fitted logistic regression models, AUC ranged between 0.57 and 0.88 (*M* = 0.72, *SD* = 0.08), indicating reasonably good predictive power [64].

## Results

### Sensitivity

For each listener and experimental block in the intensity identification task, a ROC curve was constructed from the observed rating response frequencies (for details see [65] Chapter 3). “Loud” trials on which 0.75 dB had been added to all component levels were defined as “signal”, and “soft” trials on which 0.75 dB had been subtracted were defined as “noise”. The first five trials per block were excluded from the analysis. The AUC was used as an index of sensitivity. AUC does not require strong assumptions about the internal distributions of “signal” and “noise” [50], [55]. It corresponds to the proportion of correct responses obtained with the same stimuli in a forced-choice task [66], [67] if bias-free responding can be assumed in the forced-choice task [68], [69]. To compute AUC, a maximum-likelihood procedure [70] was used for fitting a binormal model [71]. For each block, AUC and its variance were computed from the maximum-likelihood (ML) estimates of slope and intercept of the ROC curve, using the delta method [72].

Table 1 shows the average sensitivity (in terms of AUC) for each condition. As expected, the grand mean of AUC was close to 0.70 (*M* = 0.68, *SD* = 0.07). A repeated-measures ANOVA using a univariate approach and Huynh-Feldt correction for the degrees of freedom was conducted (cf. [73]), with the within-subjects variable condition (spectro-temporal condition, spectral weights condition, broadband noise condition, and the three single noise band conditions). The *df*-correction factor 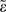 is reported. Partial *η* ^2^ is reported as a measure of effects size. The ANOVA showed a significant effect of condition, *F*(5, 45) = 19.56, *p*<0.001, 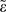 = 0.52, *η* ^2^ *_p_* = 0.69. The highest sensitivity was observed for the spectro-temporal condition. This is compatible with the theory of multiple observations [74], because in the spectro-temporal condition 30 independent components were available, while the remaining conditions contained smaller numbers of components. Post-hoc pairwise comparisons were conducted using non-pooled error terms [75] and Hochberg’s sequentially acceptive step-up Bonferroni procedure [76]. All except six tests were significant at an *α*-level of 0.05. The non-significant tests occurred for the three single-noise-band conditions versus the spectral-weights condition, the mid noise band vs. the high noise band, the low noise band vs. the broadband noise, and the spectral-weights condition versus the broadband noise.

**Table 1:** Mean sensitivity (and its standard deviation) in the intensity identification task, in terms of the area under the ROC curve (AUC), for each condition.

Condition		AUC	*SD*
Spectral weights		0.68^*^	0.04
Temporal weights	Low-CF noise band	0.67^*^	0.07
	Medium-CF noise band	0.64^*^	0.07
	High-CF noise band	0.64^*^	0.07
	Broadband noise	0.69^*^	0.06
Spectro-temporal		0.74^*^	0.07

Note. * Estimated AUC significantly different from 0.5, *p*<0.05, two-tailed.

### Spectro-temporal Weights

The normalized decision weights were analyzed via repeated-measures ANOVAs. The results are presented in the following order: 1) the results of the spectral-weights condition where the stimuli varied only spectrally, 2) the results of the conditions showing only temporal variation, and 3) the spectro-temporal weights, and, finally, 4) the weights are compared between some of these conditions.

#### Spectral weights condition

**Figure 4:**
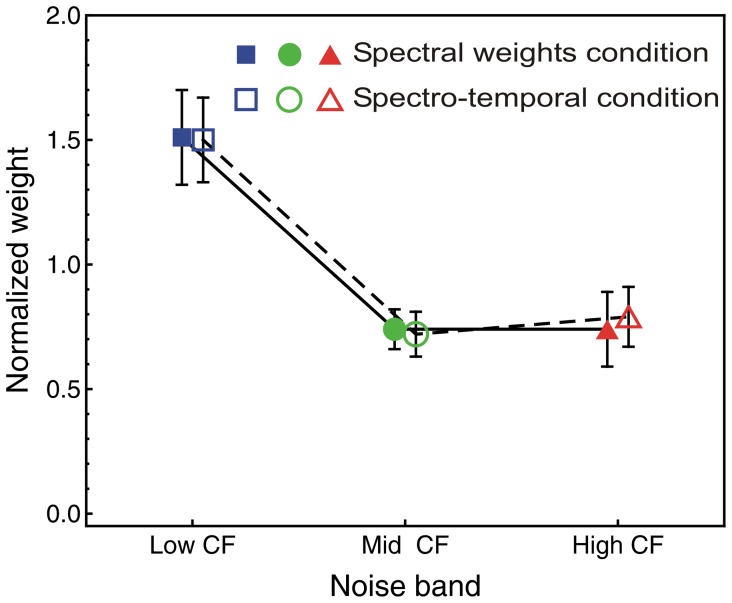
Spectral weights. Average normalized relative decision weights as a function of noise band. Normalization: mean of the absolute values of the three weights equals 1.0. Filled symbols: spectral weights condition. Open symbols: spectro-temporal condition, spectral weights averaged across segments (see text). Error bars show 95% confidence intervals.

A repeated-measures ANOVA with the within-subjects factors noise band CF (low, middle, high) and trial type (soft or loud) was conducted to test if the listeners assigned different weights to the three spectral components (noise bands) in the spectral-weights condition where there was no temporal variation. As shown by the filled squares in **Figure 4**, the low-CF noise band received a higher weight than the two other noise bands, *F*(2, 18) = 31.41, *p*<0.001, 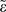 = 0.68, *η* ^2^ *_p_* = 0.78. Owing to the normalization of the weights, the effect of trial type was not significant. All interactions were also non-significant (all *p*-values >0.5).

#### Temporal weights: single-noise-band and broadband-noise conditions

**Figure 5:**
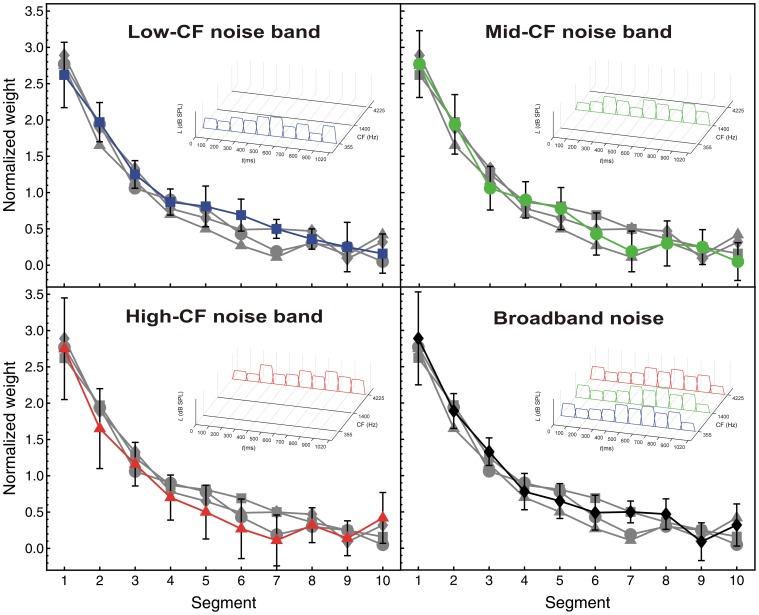
Temporal weights. Average normalized relative decision weights for the four conditions showing only temporal variation, as a function of segment number. Normalization: mean of the absolute values of the ten temporal weights equals 1.0. Blue squares: low-CF noise band. Green circles: mid-CF noise band. Red triangles: high-CF noise band. Black diamonds: broadband noise. Error bars show 95% confidence intervals.

Rennies and Verhey [31] found a significantly larger primacy effect for a broadband stimulus than for a narrowband stimulus. To test if such an effect of bandwidth was also observed in the present study an analysis was conducted of the data obtained in the broadband-noise and in the three single-noise-band conditions for which there was only temporal variation. At the same time, the question was addressed if the temporal weights differed between the low, middle and high noise bands. As seen in **Figure 5**, the weights for all of these four conditions show a clear primacy effect, because the highest weights were observed for segments 1 to 3 (i.e., the first 300 ms of the sound). There was no evidence for a recency effect. The effect of segment was significant, *F*(9, 81) = 68.71, *p*<0.001, 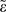 = 0.23, *η* ^2^ *_p_* = 0.88. The segment × condition interaction was not significant, however, *F*(27, 243) = 1.14, *p* = 0.33. Thus, the temporal weights did not differ between the three narrowband noises with different center frequencies, and the primacy effect was not significantly stronger for the broadband noise than for the narrowband noises. This effect is in contrast to Rennies and Verhey [31] who found a significantly larger primacy effect for broadband than for narrowband noise.

There was also a significant effect of condition, and a significant condition × trial type interaction. Despite the normalization of the weights, these effects appear because some weights with small absolute value were negative. Inspection of the individual data showed that across all listeners and conditions only three of the negative regression coefficients were significantly different from zero. Because in the normalization the mean of the *absolute values* of the weights was set to 1.0, the means were slightly lower for conditions in which negative weights occurred than for conditions where only positive weights occurred. These effects, however, have no relevance for the interpretation of the data.

#### Spectro-temporal weights condition

To our knowledge, the spectro-temporal condition including both temporal and spectral variation represents the first report of spectro-temporal weights in a loudness judgment task. Did listeners again apply non-uniform temporal or spectral weights, as in the conditions with only spectral or only temporal variation?

**Figure 6:**
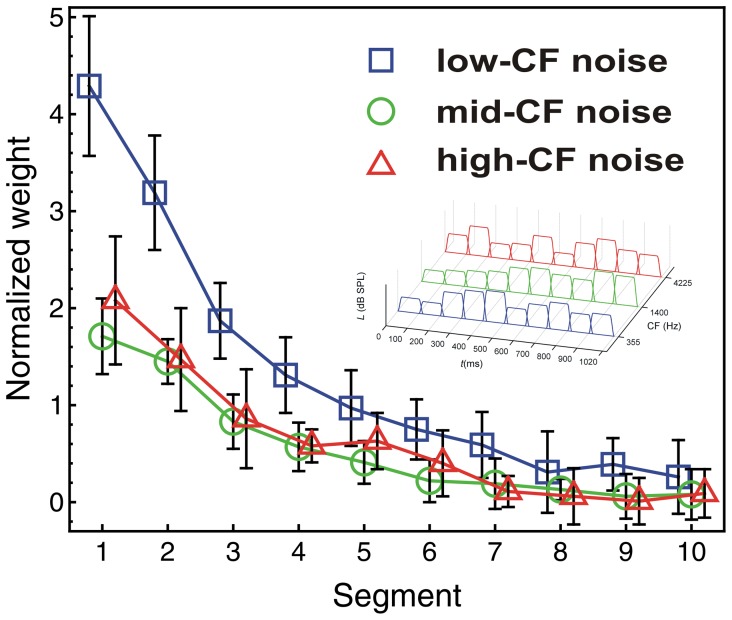
Spectro-temporal weights. Average normalized relative decision weights for the spectro-temporal condition, as a function of segment number and noise band. Normalization: mean of the absolute values of the 30 (noise band × segment) weights equals 1.0. Blue squares: low-CF noise band. Green circles: mid-CF noise band. Red triangles: high-CF noise band. Error bars show 95% confidence intervals.

A repeated-measures ANOVA with within-subjects factors segment, noise band, and trial type showed a significant effect of segment, *F*(9, 81) = 76.74, *p*<0.001, 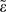 = 0.41, *η* ^2^ *_p_* = 0.90. As seen in **Figure 6**, the temporal weights for all of the three noise bands showed a clear primacy effect. The effect of noise band was also significant, *F*(2, 18) = 35.20, *p*<0.001, 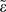 = 0.64, *η* ^2^ *_p_* = 0.80. As **Figure 6** shows, the low-CF noise band received on average higher weights than the other two noise bands, consistent with the spectral weights observed in the condition showing only spectral variation. The segment × noise band interaction was significant, *F*(18, 162) = 9.36, *p*<0.001, 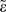 = 0.58, *η* ^2^ *_p_* = 0.51. No other effects were significant (all *p*-values >0.19).

#### Spectral weights: spectro-temporal condition versus spectral-weights condition

The above analysis showed that the spectral weights for the spectro-temporal condition followed a similar pattern as for the spectral weights condition. The low-CF noise band had a stronger influence on the loudness judgments than the two noise bands with higher CF. As stated in the introduction, an interesting question is whether the listeners applied the same spectral weights in the spectro-temporal condition as in the spectral-weights conditions. In other words, did the presence of temporal variation result in a change in the spectral weights compared to the condition without temporal variation? To answer this question, the means of the absolute values of the weights assigned to the 10 temporal segments for the low, middle, and high noise band in the spectro-temporal condition were computed for each listener and trial type. The resulting three spectral weights were then normalized so that the mean of their absolute values was 1.0. Subsequently, these spectral weights for the spectro-temporal condition were compared to the spectral weights observed for the condition where there was only spectral variation. The average spectral weights estimated for the spectro-temporal condition are shown as open symbols in **Figure 4**. A repeated-measures ANOVA with within-subjects factors condition, noise band and trial showed a significant effect of noise band, *F*(2, 18) = 37.8, *p*<0.001, 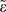 = 0.73, *η* ^2^ *_p_* = 0.81. The noise band × condition interaction was not significant, *F*(2, 18) = 0.56, *p* = 0.56. Thus, the spectral weights estimated in the spectro-temporal condition did not differ from the weights estimated in the spectral-weights condition. The remaining effects in the ANOVA were not significant (all *p*-values >0.57).

#### Temporal weights for the three noise bands: comparison between the spectro-temporal and the single-noise-band conditions

Concerning the pattern of (relative) temporal weights, two questions arise. First, did the temporal weighting patterns differ between the three noise bands in the spectro-temporal condition? Note that the ANOVA presented in the section on temporal weights does not answer this question, because the weights entering this ANOVA represented the effects of both segment and noise band. The second question is: Did the temporal weights for a given noise band estimated in the spectro-temporal conditions show a different pattern than in the corresponding single-noise-band condition?

**Figure 7:**
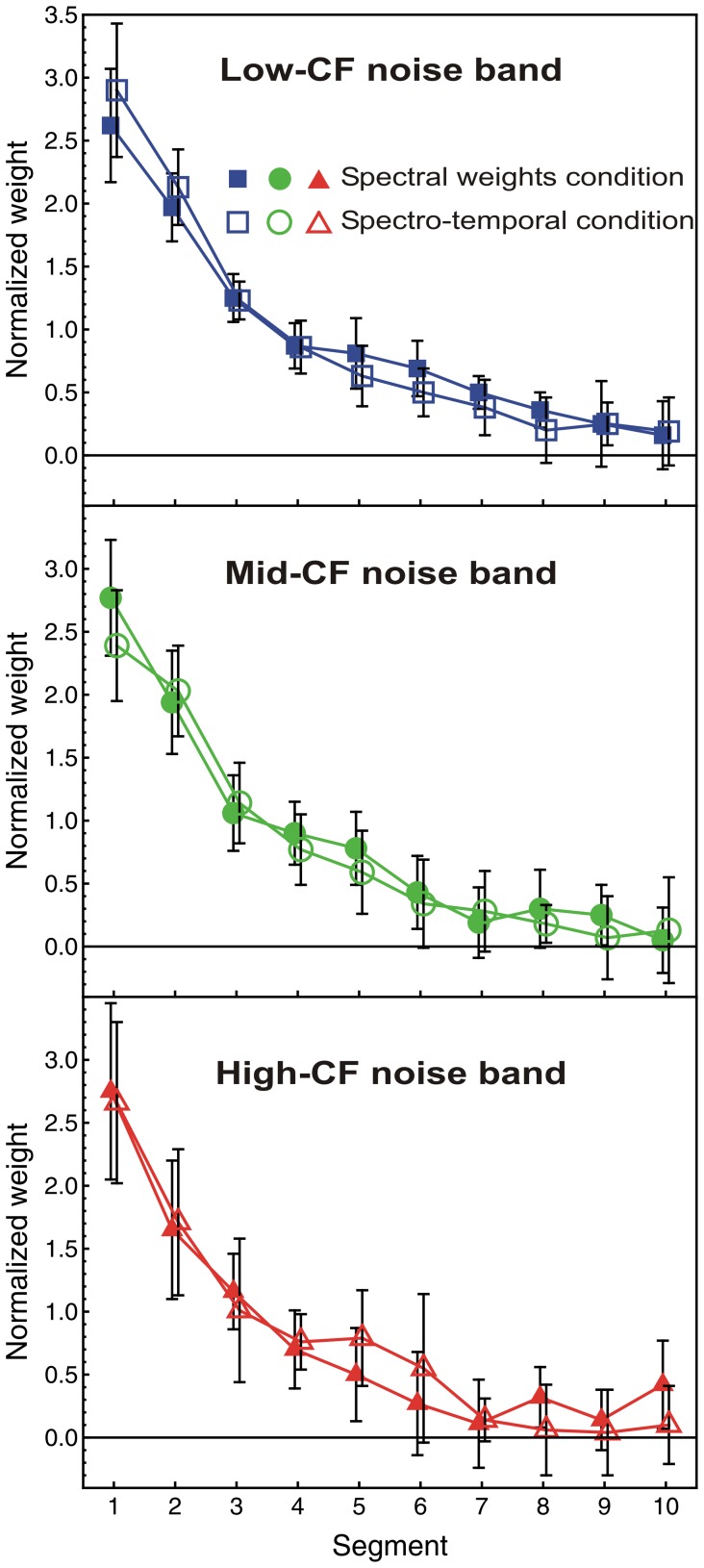
Temporal weights compared between the spectro-temporal condition and the single-noise-band conditions. Average normalized relative decision weights, as a function of segment number, condition, and noise band. Normalization: mean of the absolute values of the ten temporal weights equals 1.0 per noise band. Filled symbols: single-noise-band conditions (replotted from **Figure 5**). Open symbols: spectro-temporal condition (same data as in **Figure 6**, but different normalization). Error bars show 95% confidence intervals.

To answer these two questions, the weights in the spectro-temporal condition were normalized so that the mean of the absolute values of the 10 temporal weights was 1.0 for each listener, noise band, and trial type. The average normalized temporal weights are shown in **Figure 7** which compares the weights for the spectro-temporal condition (open symbols) to the weights for the single-noise-band conditions (filled symbols).

A repeated-measures ANOVA with within-subjects factors segment, noise band, condition, and trial type showed the expected significant effect of segment, *F*(9, 81) = 84.5, *p*<0.001, 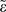 = 0.26, *η* ^2^ *_p_* = 0.90. The interactions of segment with noise band, condition, or both were not significant (all *p*-values >0.44). Thus, the pattern of temporal weights did not differ between the single-noise-band conditions and the spectro-temporal condition. There was a significant main effect of noise band, which can again be attributed to spurious negative weights.

A post-hoc ANOVA analyzing only the spectro-temporal condition also showed no significant interaction between segment and noise band, *F*(18, 162) = 0.68, *p* = 0.81. Thus, the temporal weights did not differ between frequency bands for this condition, compatible with the comparison between the temporal weights for the single-noise-band and the broadband-noise conditions presented in the section on temporal weights above.

## Discussion

Using stimuli with spectro-temporal variation in level and methods of molecular psychophysics, spectro-temporal weights in a loudness judgment task were obtained. Previous studies estimated either only temporal weights, or only spectral weights. To our knowledge, the only exception is an experiment by Dai and Berg [77]. However, they used a profile listening task where listeners detected a level increment on a single component, rather than performing a global loudness judgment task. The results of the present study demonstrate non-uniform temporal and spectral weights in the spectro-temporal condition.

### Comparison to Previous Studies on Temporal Weights for Broadband Stimuli

Several previous studies consistently showed higher weights for the first few segments of a stimulus (primacy effect). In the present experiment higher weights were found for the first three 100-ms segments. This is in the range of two to four segments found in previous studies [23], [24], [30]. As reported by Rennies and Verhey [31] and Dittrich and Oberfeld [26], the weights for the last three segments were very similar to each other. A recency effect, as found by Ellermeier and Schrödl [23] and Pedersen and Ellermeier [24], was not observed in the present study. This corroborates the conclusion that the recency effect is considerably weaker than the primacy effect [30].

It is unlikely that the primacy effect can be attributed to peripheral mechanisms like the initial peak in the firing rate of auditory nerve neurons (cf. [78]) because it is also observed for a sequence of noise bursts separated by pauses of 5, 40 or 100 ms (for a detailed discussion see [30]). With pauses of 40 or 100 ms between the sounds *each* noise burst should have elicited a similar neuronal response, due to the fast recovery of the majority of auditory nerve neurons [79], [80]. Data of Oberfeld and Plank [30] also argue against a capture of attention due to the abrupt onset of a sound [81], [82], [83] as an explanation. They attenuated the abruptness of the onset by imposing a gradual increase in level (“fade in”) across the first 300 to 700 ms of a sound with 1 s duration. This did not result in uniform temporal weights, however, but in a delayed primacy effect, with very small weights assigned to the attenuated segments constituting the fade in, and the highest weight assigned to the first unattenuated segment. Dittrich and Oberfeld [26] proposed that the primacy effect might be caused by a memory process, assuming that the levels of the different temporal portions of a sound are processed as serially sorted information, thus linking the results to experiments on working memory (e.g., [84]) and auditory sensory memory [85], where the characteristic serial position curve also showing a primacy effect is observed (for a detailed discussion see [30]). Although it may seem debatable at first sight that the individual segment levels are represented in memory, the assumption that the primacy effect can be attributed to a memory operation rather than to a mechanism specific to auditory intensity processing would be compatible with four observations. First, the primacy effect is observed for a wider range of sensory attributes like intensity (our study), frequency [29], or sound localization cues [86], [87]. This finding could be explained by a higher order mechanism like memory, although it does of course not rule out the possibility that specific mechanisms exist for intensity, frequency, localization etc. which all result in similar temporal weighting patterns. Second, the temporal weights observed for contiguous sounds as used in the present study are very similar to the weights found when the sounds are separated by pauses of 100 ms [88]. In the latter case, the stimulus is definitely broken up into perceptually distinct segments. From our own experience as listeners in the task we studied, you can clearly perceive the level changes as a sequence of events, even though the temporal segments are not separated by pauses. Thus, phenomenologically it is conceivable that the temporal portions are processed as separate events. Third, it is important to note that serial position effects like primacy−/recency-effects were not only reported for classical short term memory tasks like remembering a sequence of spoken digits, but also in *sensory memory* [85], [89]. Thus, a “symbolic” representation of the items (in the present example: segment levels) is not a prerequisite for serial position effects. Last but not least, experiments on auditory profile listening where the task was for example to detect a level increment on the temporally central sound in a sound sequence [25], [90] showed that listeners are able to selectively respond to for example a 20 ms segment embedded in a sequence of 10 contiguous segments. This result would be difficult to explain when assuming that listeners have access only to a “unitary” representation of the stimulus, and not to the separate temporal elements.

### Bandwidth Effects in Temporal Weights

Rennies and Verhey [31] argued that the higher weight assigned to the first segment may be due to spectro-temporal effects in loudness. Most studies of temporal weights used broadband stimuli [23], [24], [25], [30]. Rennies and Verhey [31] investigated how bandwidth affects the temporal weights. They found that the primacy effect is still present but is significantly reduced when a narrowband signal is used instead of a broadband signal. They argued that this effect of bandwidth may be related to the duration effect in spectral loudness summation. Several studies have indicated that the magnitude of spectral loudness summation is larger for short signals than for long signals. The stronger spectral loudness summation for short signals was attributed to slightly different auditory processing at stimulus onset compared to later portions in time [8], [9], [91], [92], resulting in a greater influence of bandwidth on loudness for short than for long signals. According to this hypothesis, the higher weight assigned to the first temporal segment of the broadband signal found by Rennies and Verhey [31] is due to increased spectral loudness summation at the beginning of the stimulus. Although a duration effect in spectral loudness summation may contribute to the primacy effect, it is unlikely that the primacy effect is solely due to this duration effect, since higher weights are also assigned to segments 2 and 3 (see **Figure 5**).

The results of the present study do not show a significantly higher weight for the first segment in the broadband condition than for the first segment in the three narrowband conditions. This difference is presumably due to differences in the stimulus parameters. Rennies and Verhey [31] used a bandpass-filtered noise with a flat spectrum geometrically centered at 2 kHz with a bandwidth of either 400 Hz (from 1810 to 2210 Hz) or 6400 Hz (from 574 to 6974 Hz). The ratio of 16:1 was larger than the bandwidth ratio of the broadband condition and the middle-band-only condition (about 8:1). It is unlikely that the loudness equalization of the bands or the spectral notch between adjacent bands caused the difference in the results between the present study and that of Rennies and Verhey [31]. Heeren *et al.* [93] found larger spectral loudness summation for a sequence of short complex tones than for long complex tones with the same spectrum, similar to the duration effect of spectral loudness for noise bursts [8], [9], [91]. Thus, the effect of duration on spectral loudness summation does not require a continuous spectrum between the lowest and highest frequency components of the stimulus.

### Comparison to Previous Studies on Spectral Weights

The results of the present study showed a higher weight on the lowest frequency band than on the higher bands (see **Figure 4**). Such a pattern was not observed in previous studies [39], [40], [41], [42], [43], with one exception [44]. The reason for the different pattern of weights observed in our study may be that the three noise bands were presented at equal loudness. In general, previous studies used the same sound pressure level for all bands. As a consequence, the bands very likely differed in loudness because absolute threshold and loudness are generally frequency dependent [94]. Below about 4000 Hz, the equal-loudness contours increase towards lower frequencies, i.e., in this frequency range, components with lower frequencies are generally softer than equal-level components at higher frequencies. If one assumes that the loudest components dominate the overall loudness, then the usage of the same SPL may have biased the weights towards the high frequencies. In line with this hypothesis Kortekaas *et al.* [40] found the highest weight for their highest frequency band, which in their study was often the loudest band (the exceptions are their complex tones with 24 components). The hypothesis is further supported by recent data from Jesteadt *et al.* [44], who found the highest weight on the lowest frequency component when each component had the same sensation level. In this case, the loudness of the *lowest* component would have been higher than that of the other components, because the slope of the loudness function is steeper at low than at high frequencies [94]. It should be noted that Jesteadt *et al.* [44] also found a slightly higher weight on the lowest band for equal-SPL (rather than equal-SL) bands. This result is difficult to reconcile with the assumption of different weights due to different component loudness. The question of whether the higher weight on the lowest band in the equal-loudness condition can be attributed to the frequency-dependent slope of the loudness function is discussed below (section *Spectro-temporal weights within loudness models*).

### Prediction of Spectro-temporal Weights from Temporal and Spectral Weights

The analyses presented above show a strong similarity between the average spectral and temporal weights obtained, one the one hand, with the less complex stimuli (i.e., with either only spectral or only temporal variability) and, on the other hand, with the stimuli in the spectro-temporal condition. A simple but strong hypothesis concerning the spectro-temporal weighting of loudness compatible with these results is that the spectral weights do not change as a function of time (i.e., the same spectral weights apply for each temporal segment), and that the temporal weights do not change across frequency (i.e., for each frequency band the same temporal weights apply). If this hypothesis were correct, it would be possible to predict the spectro-temporal weights for each listener by multiplying the spectral weights (estimated in the *spectral-weight condition*) with the temporal weight for each temporal segment (estimated in the *broadband-noise condition*). In more formal terms, the component weights in Eq. (1) (e.g., *w_nbL,i_*) should be given by
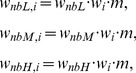
(3)where *w_nbL_*, *w_nbM_*, and *w_nbH_* are the spectral weights for the low-CF, middle-CF, and high-CF noise bands, respectively, as estimated in the spectral weight condition, and *w_i_* is the temporal weight for segment *i* estimated in the broadband noise condition. The constant *m* was included because the spectral and temporal weights entering Eq. (1) are relative weights and therefore specify the component weights (i.e., regression coefficients) only up to a multiplicative constant.

To test this hypothesis, the individual spectral and temporal weights (as estimated in the spectral weight condition and the broadband noise condition, respectively) were entered into a logistic regression model analyzing the responses from the spectro-temporal condition. As the trial type (“soft” or “loud”) had no effect on the weights in the above analyses, the arithmetic mean across the two trial types was used. Thus, for each listener, there were three spectral weights (*w_nbL_*, *w_nbM_*, and *w_nbH_*) and ten temporal weights (*w*_1_ to *w*_10_). For a given trial from the spectro-temporal condition, a weighted average *D_pred_* of the 30 component levels presented in this trial was computed, combining Eq. (1) and Eq. (3). Subsequently, a logistic regression model was fitted relating the response to *D_pred_*. This model had four free parameters: one regression coefficient corresponding to *m* in Eq. (3), and three intercepts corresponding to the *c_j_* in Eq. (1). For each listener and trial type, the global goodness-of-fit (log likelihood) of this restricted model was compared to the goodness-of-fit of the full model in which the 30 component weights were estimated from the data obtained in the spectro-temporal condition. The full model had 33 free parameters (30 component weights plus 3 intercepts). A likelihood-ratio test was used for model comparison. As the full model has 29 more free parameters than the restricted model, the test statistic is distributed as *χ* ^2^(29). For 10 of the 20 (subject × trial type) conditions, the fit of the full model was not significantly better than the fit of the restricted model (*p*>0.05). The average predictive power as indexed by AUC was 0.68 (*SD = *0.05) for the model based on *D_pred_*, which still represents an acceptable quality of the predictions but was significantly smaller than the AUC for the full model (*M* = 0.72, *SD* = 0.08), *F*(1, 9) = 75.9, *p*<0.001. Thus, the 30 component weights for the spectro-temporal condition estimated from the trial-by-trial data for each trial type provided a better fit than the component weights predicted from the spectral and temporal weights (Eq. (3)). The latter model, however, still provided a surprisingly good fit if one considers that it has 29 fewer free parameters than the full model. It can therefore be concluded that the spectral weights remain constant across time, and the temporal weights do not change across frequency. This pattern of result suggests that the temporal and the spectral weighting of the loudness of a time-varying sound are independent processes. One should note that this may not be true for all stimuli. The bandwidth-dependent weights found by Rennies and Verhey [31] are not consistent with independent processes. Further studies are needed to investigate for what type of stimuli this assumption is valid.

### Spectro-temporal Weights within Loudness Models

As mentioned above, the slope of the loudness function is generally steeper at low than at intermediate and higher frequencies. Suzuki and Takeshima [94] estimated the slope of the loudness function, i.e. the compressive exponent in the power law relating loudness and intensity to be about 0.3 at 1 kHz and 4 kHz, but about 0.4 at 125 Hz (see their Fig. 7). The relation between the frequency-dependent slope of the loudness function and the spectral weights is consistent with the measured sensitivity for the three single-noise-band conditions. As discussed in section *Sensitivity*, in those conditions AUC was significantly higher for the noise band with low CF than for the two other noise bands (see Table 1).

**Figure 8:**
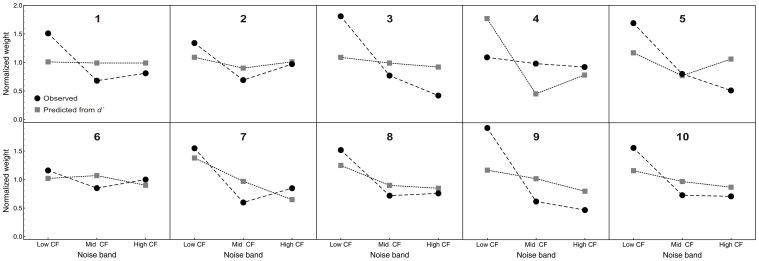
Individual spectral weights: observed versus predicted. Average normalized relative decision weights, as a function of noise band. Black circles: weights estimated from the spectro-temporal condition. Gray squares: weights predicted from the sensitivity in the single-noise- band conditions (see text).

The optimal decision rule would be to apply spectral weights proportional to sensitivity for the three bands, as indexed by *d* ´ (integration model; [95]). If one follows this rationale, do the observed differences in sensitivity between the three noise bands account for the observed differences in the spectral weights? To answer this question, *d* ´ observed in the three single-noise-band conditions (Table 1) was individually normalized to an arithmetic mean of 1.0 across the three noise bands. These normalized *d* ´ values represent the weights predicted by the integration model. **Figure 8** plots the observed and predicted spectral weights for each listener. While the observed and predicted weights show rather good agreement for some listeners, there are considerable deviations for other listeners, and in most cases the predicted weights show smaller variability than the observed weights. To quantify the relation between the observed and predicted weights, the correlation coefficient within listeners was computed [96]. This correlation coefficient provides information about whether an increase in one variable (predicted weight) within the individual is associated with an increase in the other variable (observed weight). The data showed a significant but weak correlation between the observed and predicted weights, *r* = 0.44, *p* = 0.039. Thus, the differences in sensitivity to changes in the level of the three noise bands can partially, but not completely, account for the spectral weights applied by the listeners.

A second interesting question concerning the influence of the slope of the loudness functions on the present data is whether the observed spectral weights can be derived directly from the frequency-dependent exponents. If one assumes that the global loudness is the sum of the component loudnesses (i.e., the component levels in Eq. (1) are replaced by component loudnesses), then a level change by for example 1 dB would result in a stronger change in overall loudness when imposed on a stimulus component with a steeper loudness function. As a consequence, the analysis we used would show a higher regression coefficient for a component with a steep slope of the loudness function, that is, a higher relative weight. To answer the question of whether the spectral weights can be explained by the slopes of the loudness function, a power law according to Stevens [97], [98] was used, representing the most basic loudness model:
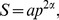
(4)where *S* denotes the perceived loudness, *p* is sound pressure, *a* is a dimensional constant, and *α* is the compressive exponent, i.e. the slope of the loudness function. To focus this analysis purely on the role of the exponent *α*, any influence of bandwidth was neglected, i.e., the values of *a* and *α* as derived for pure tones by Suzuki and Takeshima were used [94].

The values of *α* for the low, middle, and high center frequencies of the stimuli used in the present study were taken directly from Fig. 7 of [94] and were 0.335, 0.293, and 0.289, respectively. The equal-loudness contours shown in Fig. 11 of [94] were used to equalize the loudness of the three frequency components, when the mid-CF component had a level of 55 dB SPL as in the experiment. The corresponding levels of the low- and high-CF components were 57.0 and 51.8 dB SPL. These levels were then used to calculate the dimensional constant *a* for each CF, such that finally one equation of the form of Eq. (4) was available for each frequency component. Subsequently, 5000 random levels were generated for each frequency component using mean values of 57.0, 55.0, and 51.8 dB SPL, a standard deviation of 2.5 dB, and random level increments of +0.75 or −0.75 dB. In other words, the levels were generated exactly as in the experiment except that the frequency components were equalized in loudness based on the equal-loudness contours given in [94]. For each component, the loudness was calculated using the power law described above, and the overall loudness was calculated as the sum of the three component loudnesses. This simplifying assumption concerning loudness summation seemed justified because of the relatively wide spacing of the three components [12]. The overall loudness was then used to estimate spectral weights using the same logistic regression procedure as for the data analysis, with a simulated decision criterion corresponding to the arithmetic mean of overall loudness across the 5000 simulated trials. After normalizing to a mean weight of 1.0, this resulted in estimated spectral weights of 1.10, 0.96, and 0.94 for the low-CF, mid-CF, and high-CF components, respectively. In agreement with the data, the weight assigned to the lowest frequency component was higher than for the other frequencies, while the weights on the mid- and high-CF components were similar. However, the difference between the three weights was much smaller than observed in the data (see **Figure 4**). Thus, the frequency-dependence of slopes of the loudness functions can only partially predict the observed spectral weights. This conclusion is consistent with data of Leibold *et al.* [42] who measured spectral weights for five-tone complexes with different bandwidths. For bandwidths smaller than 500 Hz, the bowl-shaped spectral weights were rather accurately predicted by the loudness model of Moore *et al.* [10]. However, for a bandwidth of 2119 Hz (which is smaller than the 5100 Hz bandwidth we used), the model predicted almost uniform spectral weights, while the observed weights showed a much higher weight on the highest component than on the other components. Leibold and co-authors suggested that at smaller bandwidths the components mask each other, and that this peripheral interaction between adjacent components is well represented in the loudness model which is based on excitation patterns. In contrast, at the highest bandwidth peripheral interactions should be negligible, so that the non-uniform spectral weights observed for the 2119 Hz bandwidth can be attributed to a more central effect. Similar results were reported by Leibold *et al.* [41] for a broadband tone complex.

Additional simulations showed that when the three components were identical in *level* (55 dB SPL) instead of being identical in loudness, the estimated normalized weights were 0.87, 0.91, and 1.22. Thus, at equal SPL the highest weight is predicted for the high-CF component. As discussed above, this is consistent with the results of several studies which presented the different frequency components at equal SPL [39], [40], [41].

An interesting extension of the present work would be to measure individual loudness functions for single components and spectral weights in the same listeners and to directly analyze the predictability of spectral weights from the measured exponents. Such an analysis could provide a closer view on the contribution of differences in slope to the spectral weights and how individual differences in the slope are reflected in individual differences in the weights. However, the analysis presented above showed that, on average, the higher weight observed for the low-CF component can only partially be attributed to the frequency-dependent slope of the loudness function. Thus, it remains for future research to fully understand the reason for this non-uniform spectral weighting pattern.

At this point, it is important to recall that the weights represent the influence of changes in the level of a specific component on the *global* loudness of the multi-component stimulus. These weights will not necessarily be identical to the slope of the loudness function for a component presented *in isolation*. As an example, consider one of the single-noise-band stimuli (Figures 3 and 5). For example, the slopes of the “isolated” loudness functions are identical for segments 2 and 9, because these components are identical in spectrum, duration, and average level. However, the weights assigned to the two components differ considerably. In other words, the loudness function for a component presented *in isolation* is not identical to the slope of the function relating the component level and the *global* loudness of the multi-component stimulus. The same conclusion can be drawn from the above simulation which demonstrated that the spectral weights cannot be explained by the slope of the loudness function for the three frequency bands presented in isolation.

### Summary and Conclusions

A limitation of previous studies concerned with the decision weights listeners apply when judging the loudness of complex sounds is that they either considered the *temporal* weighting of loudness but did not look at *spectral* weights, or measured spectral weights but did not consider temporal aspects of loudness. The present study took the investigation of the loudness of complex, dynamic stimuli one step further and estimated *spectro-temporal weights* for global loudness judgments by introducing independent temporal variations in level in different spectral regions within each stimulus. The analyses, based on methods of molecular psychophysics, showed that for stimuli which change in spectral composition across time listeners place a higher weight on the lowest frequency component. The temporal weights showed a clear primacy effect, that is, a stronger influence of the sound energy at the beginning of the sound than of the level of later temporal portions. Comparisons of these spectral and temporal weights to weights obtained in control conditions with only temporal or only spectral variation were used to answer the question whether the spectro-temporal weights can be individually predicted from spectral and temporal weights. These analyses showed that this is possible at a rather high precision, indicating that the temporal and the spectral weighting of the loudness of a time-varying sound are independent processes. The spectral weights remain constant across time, and the temporal weights do not change across frequency. The observed non-uniform spectral weights cannot fully be accounted for by loudness models based on the frequency-dependent slope of the loudness function. This observation is compatible with previous suggestions that central rather than peripheral processes are responsible for the observed spectral and temporal weights. These are not yet implemented in current loudness models. Additional research is necessary here.
